# Annotations

**Published:** 1895-06-29

**Authors:** 


### ANNOTATIONS.

### The Doctor's Twopence.

DR. R. JONES, of Leigh, is evidently a humorous man. He has instituted a new method of dealing with casual calls on his professional charity, and flies to the New Testament for a defence of his position. If any person, he tells us, whether man, woman, or child, is taken ill within hail of a doctor's house, a dozen eager philanthropists rush off to secure the doctor's aid, and nothing will satisfy their enthusiasm of sympathy but that the doctor must go with them at once. Very well, says the doctor; but if you are so kind and charitable, are you prepared to pay my fee? Are you going to be sympathetic to the sick person at your own expense, or at mine? If the caller demurs to this view of the case the doctor is ready with a Scripture parable for him. "A certain man, going down from Jerusalem to Jericho, fell among thieves; and, after being roughly handled by them, was left half dead. What the Priest and the Levite did is known to all men. What the Good Samaritan did is also known, but it is only imperfectly understood. He put the injured man on his own beast, he took him to an inn, and he paid twopence to the landlord beforehand." This is the point, Dr. Jones contends, which is overlooked by our modern Good Samaritan. He would pick up the injured man, and put him on his own beast, and carry him to an inn, right zealously. But once there, he would expect the landlord to be responsible for any further expense. That, at any rate, is what the Good Samaritan invariably does to the doctor. Now, why should the doctor give his time, skill, and physic to an injured person for nothing, any more than the hotel-keeper should give lodging and food? The question, it seems to us, is entirely pertinent. Hunger is as dangerous to life as illness. Yet no person who wishes to be kind to a hungry man takes him to the door of the baker's shop, expecting the baker to feed him. No; he pays the baker for a loaf. Dr. Jones says he now always declines to attend casual cases unless the person who calls him first pays his fee. "If you are a Good Samaritan," he says in effect, "where is your twopence?"

### "Egomania."

WE cannot quite agree with Dr. Giovanni Mingazzini, of the University of Rome, that Lord Byron was an egomaniac, though it must be admitted that the cumulative evidence collected by the Roman doctor makes out a case which is all but entirely convincing. Byron was not mad. On the contrary, if he had been well whipped as a boy on fitting occasions, and well instructed and disciplined always, he might have displayed all the genius he did in fact, display, and a good deal more, without any of the freaks and absurdities which give plausibility to the charge of madness which has so frequently been brought against him. According to new teaching, the man or woman who does not loyally work at some occupation in which the "ego" may be forgotten for a certain number of hours every day, but who is always and for ever thinking of and planning for self, is neither more nor less than a person of unsound mind. Carrying this sound principle still further, it is held that the man or woman who writes books—novels, stories, poetry, and the like—in which this excessive study of, and devotion to self is held up to admiration, and idealised, is also a lunatic. Scientific medicine owes a great duty to the public in this connection. It sees with absolute clearness that excessive devotion to self is a true form of mania, and it gives the mania a name—"egomania." But it must go further; it must teach the unscientific world incessantly that this form of mania, when it lays hold of clever persons who can write, is dangerous to others by the hold these books gain over their minds.

### A Good Example.

IN no year since the establishment of the Hospital Sunday Fund in London has its annual collection been a matter of such universal interest. The results, however, which must follow from the wide circulation of the facts and figures in the Special Supplement of the 15th inst. will not be limited to the present collection. New interest thus aroused must have time to fructify, and later may come the harvest. Most numerous and gratifying have been the expressions of interest and sympathy and desire to co-operate, which have found their way to our letter box. From Admiral Ward comes a letter striking a keynote which should find response and echo throughout the metropolis. If it does the goal will be reached, and hospital appeals in the coming year must prove more fruitful than ever before. "If the sums collected in the churches and elsewhere do not reach the anticipated sum of £70,000, I will double the subscription I gave yesterday in church," writes Admiral Ward, words which afford justsuch a practical example as we could most desire. Could Admiral Ward's words animate every well-to-do Londoner, there would be assurance that what the sick of our great city need would be forthcoming. With a zeal that calls for admiration the clergy have proved themselves leaders in the work of mercy, and all that eloquence could do in the good cause was done on Hospital Sunday. The Church of England showed herself above all others the Church of the people. Her ministers called for, and her congregations gave in greater proportion than any other denomination. One correspondent especially desired us to point this out, and we willingly do so, for the encouragement of the clergy and their congregations, and with the hope of stimulating a laudable competition amongst the followers of other creeds. All Christian hands should surely glory in uniting to help suffering which is relieved independently of difference in religion. Burdett's "Hospital Annual" shows that last year Hospital Sunday reaped £28,368 in collections from the Church of England, under £4,000 from the Nonconformists, and but £484 from the Roman Catholic churches. This year there is a general increase in the collections. But a few exceptions are apparent. One clergyman of a church in a fashionable part of London writes that whilst "the contents of your Supplement largely helped to stir people's feelings" many of his richest parishioners were absent from London. To him, and to other clergy in the same position, we have pointed out that copies of our Supplement, with its appeal, are at their disposal to assist in bringing to the Mansion House the gifts of those donors who were unable to give in person. If all give to the extent of their power, there will be no need to appeal further or to remind Admiral Ward of his most encouraging promise.

